# Machine learning techniques for arrhythmic risk stratification: a review of the literature

**DOI:** 10.1186/s42444-022-00062-2

**Published:** 2022-04-01

**Authors:** Cheuk To Chung, George Bazoukis, Sharen Lee, Ying Liu, Tong Liu, Konstantinos P. Letsas, Antonis A. Armoundas, Gary Tse

**Affiliations:** 1 Cardiac Electrophysiology Unit, Cardiovascular Analytics Group, China-UK Collaboration, Hong Kong, China; 2 Department of Cardiology, Larnaca General Hospital, Larnaca, Cyprus; 3 Medical School, University of Nicosia, Nicosia, Cyprus; 4 Heart Failure and Structural Cardiology Ward, First Affiliated Hospital of Dalian Medical University, Dalian, Liaoning Province, China; 5 Tianjin Key Laboratory of Ionic-Molecular Function of Cardiovascular Disease, Department of Cardiology, Tianjin Institute of Cardiology, Second Hospital of Tianjin Medical University, Tianjin 300211, China; 6 Department of Electrophysiology, Onassis Cardiac Surgery Center, Athens, Greece; 7 Cardiovascular Research Center, Massachusetts General Hospital, 149 13th Street, Charlestown, Boston, MA 02129, USA; 8 Institute for Medical Engineering and Science, Massachusetts Institute of Technology Cambridge, Cambridge, MA, USA; 9 Kent and Medway Medical School, Canterbury, Kent, UK

**Keywords:** Artificial intelligence, Machine learning, Ventricular arrhythmias, Ventricular tachycardia, Ventricular fibrillation, Risk stratification, Prediction models

## Abstract

Ventricular arrhythmias (VAs) and sudden cardiac death (SCD) are significant adverse events that affect the morbidity and mortality of both the general population and patients with predisposing cardiovascular risk factors. Currently, conventional disease-specific scores are used for risk stratification purposes. However, these risk scores have several limitations, including variations among validation cohorts, the inclusion of a limited number of predictors while omitting important variables, as well as hidden relationships between predictors. Machine learning (ML) techniques are based on algorithms that describe intervariable relationships. Recent studies have implemented ML techniques to construct models for the prediction of fatal VAs. However, the application of ML study findings is limited by the absence of established frameworks for its implementation, in addition to clinicians’ unfamiliarity with ML techniques. This review, therefore, aims to provide an accessible and easy-to-understand summary of the existing evidence about the use of ML techniques in the prediction of VAs. Our findings suggest that ML algorithms improve arrhythmic prediction performance in different clinical settings. However, it should be emphasized that prospective studies comparing ML algorithms to conventional risk models are needed while a regulatory framework is required prior to their implementation in clinical practice.

## Introduction

Fatal ventricular arrhythmias (VAs) and sudden cardiac death (SCD) are some of the most important study outcomes in the field of cardiology. Current efforts have focused on the prediction of VAs in different diseases, including hypertrophic cardiomyopathy, arrhythmogenic cardiomyopathy, heart failure (HF), congenital heart diseases, cardiac ion channelopathies, in addition to the risk of VAs among the general public [1–6]. Conventional risk scores are the most widely used tools for risk stratification purposes in clinical practice [7]. However, these risk scores have several limitations, including variations among validation cohorts, the inclusion of a limited number of predictors while omitting some variables that might be important. As a result, clinical scores that can accurately predict major outcomes and therefore can aid in personalized clinical management are needed.

Machine learning (ML) can integrate and interpret data from different domains in settings where conventional statistical methods may not be able to perform [8]. Recently, the role of ML techniques has been studied in different aspects of medicine, including electronic health records, diagnosis, risk stratification, timely identification of abnormal heart rhythms in the intensive care unit [9, 10], on prognosis and guidance of personalized management [11, 12]. However, application of ML study findings has been limited due to the lack of a regulatory framework for its implementation and the clinicians’ unfamiliarity in using as well as trusting ML techniques [13]. This review aims to present existing data regarding the role of ML techniques in the risk stratification of VAs in different clinical settings.

## Machine learning algorithms

ML algorithms can aid the interpretation of complex data, stratification of patient diagnosis and delivery of personalized care and therefore are particularly useful in the management of cardiovascular diseases [8]. Random forest, convolutional neural network (CNN) and long short-term memory network (LTSM) models are three commonly used ML approaches in cardiovascular medicine.

### Random forest model

**Fig. 1:**
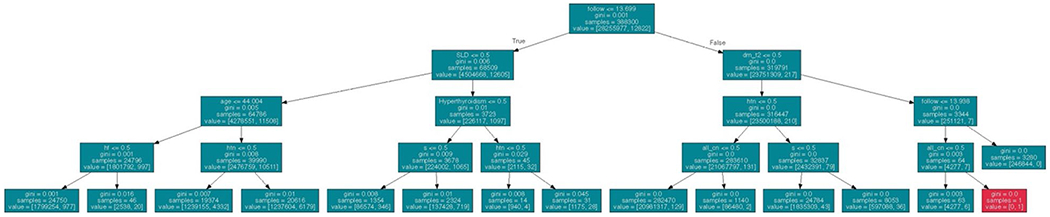
First decision tree of the random forest model predicting risk of atrial fibrillation [15]

One common model ensemble method is known as the random forest model. The underlying idea of random forest algorithms originates from the assumption that predictions derived from a large ensemble of models are more accurate and robust compared to using a single model. Within a random forest model, numerous decision trees form the basic building blocks by performing either classification or regression tasks. To classify the data, it is processed through a series of true or false questions, allowing information to be categorized into the purest possible subgroups. Each decision tree will then classify a new object based on specific attributes through voting, and the classification is based on the largest sum of votes. In the case of regression, the average outputs from different trees are calculated [14]. With this algorithm, large data sets with higher dimensionality can be processed. When a group of uncorrelated decision trees in collaboration can reduce the effect of individual variability and errors, thus outperforming constituent trees [14]. There are two main ML ensemble meta-algorithms to ensure the trees are uncorrelated: bagging and featuring randomness. The former method separates the data into small subsets via random sampling with replacement, improving the stability of ML; at the same time, the latter shuffles specific features of the data set, increasing the diversity in trees. Due to the simplicity of individual trees, this lowers the training time and can be applied to academia, e-commerce and banking sectors. An illustration of one decision tree in a random forest model used to predict atrial fibrillation can be shown in Fig. 1 [15]. The model was trained with a sample of 682,237 Chinese subjects. In each decision tree, there was a maximum depth of four nodes. Any greater than four nodes in each tree were found to cause overfitting.

### Convolutional neural networks

**Fig. 2:**
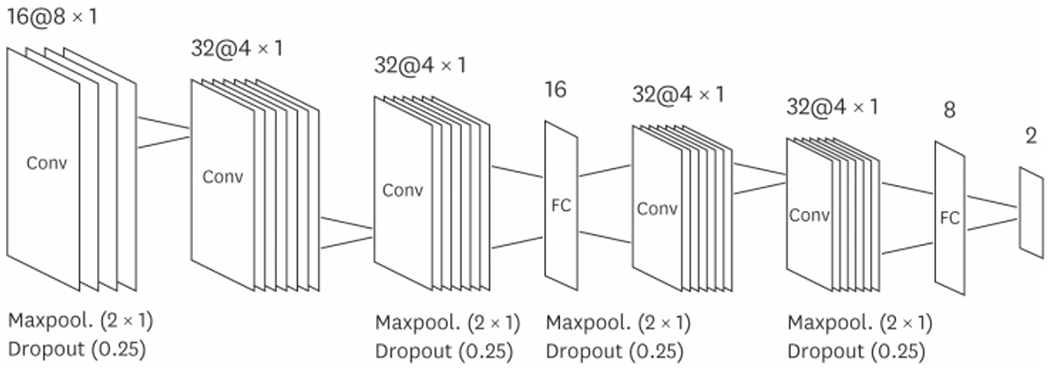
Seven-layered optimal architecture of the CNN model predicting risk of atrial fibrillation [16]

Convolutional neural networks (CNNs) are used to detect patterns and to classify images with a high level of precision using filters. Different types of filters detect various forms of patterns depending on their level of sophistication. Pattern detection can range from simple geometric shapes to complex objects such as eyes and dogs. The main purpose of a CNN is to receive and transform an input through a convolutional operation. In a convolutional operation, the process requires an input image, feature detector and feature map [14]. A feature detector consists of a matrix. The matrix can contain any digits corresponding to a specific color or feature that is being measured. This detector is placed over the input image, and the number of cells that match between the feature detector and the image is counted pixel by pixel. Often, CNN analysis may break down an image into smaller parts for higher precision during matching. After a series of calculations, this generates a feature map that indicates where a specific feature occurs. This process of convolving and filtering an image to generate a stack of filtered images is known as a convolution layer [14]. Realistically, for CNN to generate practical data, this would require multiple feature detectors to develop multiple feature maps. Following on, the output is passed onto an adjoining layer. This process is repeated until it reaches the final layer known as a fully connected layer, where a list of featured values converts into a list of votes for a category. Through training, CNN can prioritize the detection of features in chronological order with higher accuracy. An example of an optimal architecture of a CNN model used to predict atrial fibrillation can be demonstrated in Fig. 2 [16]. A one-dimensional convolution was used in the convolution layer as electrocardiographic (ECG) signals are also a one-dimensional time series. Other functions including dropout, batch normalization and a rectified linear unit were also included to prevent divergence.

### Long short-term memory network

**Fig. 3:**
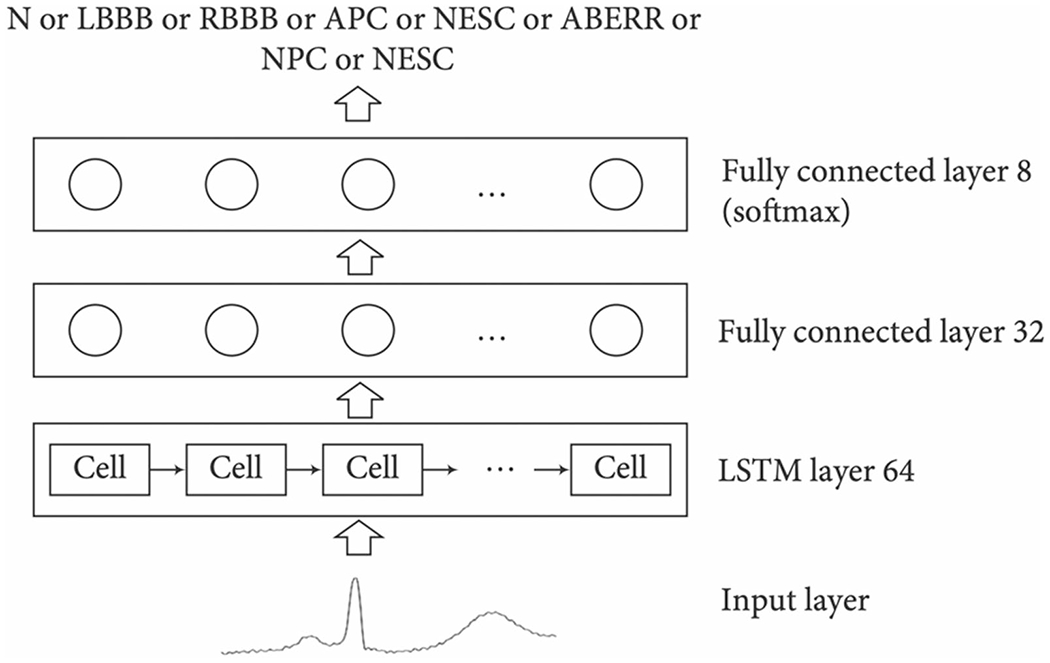
LSTM recurrent network architecture detecting arrhythmia on imbalanced ECG datasets [17]

Long short-term memory network (LTSM) is a type of gated recurrent neural network that regulates the flow of information. Hence, this allows the algorithm to learn to differentiate between unnecessary and relevant information for making predictions. The process begins with transforming a sequence of words into machine-readable vectors. These vectors are processed by transferring the previous hidden state into the next cell, which includes learned information from the previous network. Within an LSTM unit cell, there are three gates: The input gate controls whether the memory cell is updated, the output gate controls the visibility of the current cell state, and the forget gate ensures the memory cell is reset to 0. The previous and current inputs are combined to form a vector, which then goes through Tanh activation. Through a series of complex calculations and processing, LSTM can learn long-term dependencies. An example of an LTSM recurrent network architecture with focal loss, used to detect arrhythmia, is shows in Fig. 3 [17]. The four-layered LTSM network was designed to decipher the timing features in complex ECG signals, which is coupled with the focal loss to fix category imbalance. Epochs were set to 350 to achieve stability in classification accuracy.

## Specific patient populations

ML algorithms have been used for arrhythmic risk stratification purposes and can provide an incremental value for the risk stratification of cardiomyopathies (Table 1).

### Hypertrophic cardiomyopathy

A simple clinical score is recommended according to the current guidelines for the VA risk stratification of patients with hypertrophic cardiomyopathy (HCM) [18]. However, the analysis and implementation of more variables for VA risk stratification purposes seem to improve the predictive accuracy in this population. Specifically, the application of ML methods to electronic health data has identified new predictors of VAs in this population, while the ML-derived model performed better compared to current prediction algorithms [19]. In another study, the ensemble of logistic regression and naïve Bayes classifiers was most effective in separating patients with and without Vas [19]. A recent study proposed a novel ML risk stratification tool for the prediction of five-year risk in HCM, which showed a better performance compared to conventional risk stratification tools regarding SCD, cardiac and all-cause mortality, while the best performance was achieved using boosted trees [20]; specifically, the authors used demographic characteristics, genetic data, clinical investigations, medications and disease-related events for risk stratification purposes.

Cardiac magnetic resonance (CMR) has been found to provide important data for risk stratification purposes in HCM patients [21]. Of the studied CMR indices, late gadolinium enhancement (LGE) has a major role in the risk stratification of this population [22]. The extent of LGE has been found to outperform current guideline-recommended criteria in the identification of HCM patients at risk of Vas [23]. In this context, ML techniques can improve the accuracy in the identification of high-risk patients. Specifically, ML-based texture analysis of LGE-positive areas has been proposed as a promising tool for the classification of HCM patients with and without ventricular tachycardia (VT) [24]. In this study, of eight ML models investigated, k-nearest-neighbors with synthetic minority oversampling technique depicted the best diagnostic accuracy for the presence or absence of VT [24].

12-lead Holter ECGs have also been analyzed using mathematical modeling and computational clustering to identify phenotypic subgroups of HCM patients [25]. Specifically, using these methods, it has been found that HCM can be classified into patients with T-wave inversion with and without secondary to QRS abnormalities. HCM patients with T-wave inversion not secondary to QRS abnormalities have been associated with an increased risk of SCD [25].

### Other cardiomyopathies

The risk stratification of fatal arrhythmias is also significant in myocardial infarction patients. CMR has also been found to provide incremental data for risk stratification purposes in this population [26]. Quantitative discriminative features extracted from LGE in post-myocardial infarction patients have been studied for the discrimination of high- versus low-risk patients. In a study, the leave-one-out cross-validation scheme was implemented to classify high- and low-risk groups with a high classification accuracy for a feature combination that captures the size, location and heterogeneity of the scar [27]. Furthermore, nested cross-validation was performed with k-neural network, support vector machine, adjusting decision tree and random forest classifiers to differentiate high-risk and low-risk patients. In this context, the support vector machine classifier provided average accuracy of 92.6% and area under the receiver operating curve (AUC) of 0.921 for a feature combination capturing location and heterogeneity of the scar [27].

Recently, a novel ML approach was studied for quantifying the three-dimensional spatial complexity of grayscale patterns on LGE-CMR images to predict VAs in patients with ischemic cardiomyopathy [28]. Specifically, in this study, a substrate spatial complexity profile was created for each patient. The ML algorithm was classified with 81% overall accuracy, while the overall negative predictive value was estimated at 91% [28]. The clinical importance of these findings is mainly attributed to the high negative predictive value of the method that can identify ischemic cardiomyopathy patients who will not be benefited from an implantable cardioverter-defibrillator (ICD).

Except for clinical and imaging variables, cellular electrophysiological characteristics have also been studied using ML algorithms to identify ischemic cardiomyopathy patients at risk of SCD [29]. ML of monophasic action potential recordings in ischemic cardiomyopathy patients revealed novel phenotypes for predicting sustained VT/fventricular fibrillation (VF) [29].

Another clinical entity that needs a better risk stratification tool is HF with reduced ejection fraction (HFrEF) due to non-ischemic dilated cardiomyopathy. While previous studies had proved the benefit of ICDs in the primary prevention of SCD in this setting [30], the Danish Study to Assess the Efficacy of ICDs in Patients with Non-ischemic Systolic Heart Failure on Mortality (DANISH) trial showed that prophylactic ICD implantation in patients with symptomatic systolic HF that not caused by coronary artery disease was not associated with a significantly lower rate of all-cause mortality [31]. It is of great clinical importance to identify those patients with HFrEF due to non-ischemic etiology, who will benefit from an ICD. ML techniques can play a crucial role in better stratifying this group of patients [32].

In a recent study, ML techniques were used to identify cardiac imaging and time-varying risk predictors of appropriate ICD therapy in HFrEF patients [33]. It was found that baseline CMR imaging metrics (specifically, left ventricle heterogeneous gray and total scar, left ventricle and left atrial volumes, and left atrial total emptying fraction) and interleukin-6 levels were the strongest predictors of subsequent appropriate ICD therapies [33]. It is well known that ICD shocks have been associated with adverse events in patients with ICDs [34]. ML techniques and specifically random forest have been used for the prediction of short-term risk of electrical storm in patients with an ICD using daily summaries of ICD measurements [35]. The clinical importance of these methods can be mainly attributed to the preventive measures that can be adopted to avoid an imminent arrhythmic event. ML algorithms can further improve the existing risk stratification tools, and the ML-derived models can help clinicians to optimize the management of HFrEF patients.

Non-compaction cardiomyopathy is another clinical setting that needs further research for better characterization and risk stratification. In a recent study, the presence of significant compacted myocardial thinning, an elevated B-type natriuretic peptide or increased left ventricular dimensions were significantly associated with adverse events in non-compaction cardiomyopathy patients [36]. ML techniques have been implemented for improving risk stratification in these patients. Specifically, echocardiographic and CMR data were analyzed using ML algorithms to identify predictors of adverse events in non-compaction cardiomyopathy patients [37]. The combination of CMR-derived left ventricular ejection fraction, CMR-derived right ventricular end systolic volume, echocardiogram-derived right ventricular systolic dysfunction and CMR-derived right ventricular lower diameter was found to achieve the better performance in predicting major adverse events in these patients [37].

### Sarcoidosis

Cardiac sarcoidosis is another clinical condition that mandates a better arrhythmic risk stratification model given the increased risk of complete heart block, VA and SCD. Currently, an ICD should be considered in patients with atrioventricular block requiring pacemaker implantation independently of the left ventricular ejection fraction [38].

ML techniques have been used for diagnosing and optimizing the arrhythmic risk stratification of these patients. While the ^18^F-fluorodeoxyglucose (18F-FDG) positron emission tomography (PET) plays a critical role in the diagnosis of cardiac sarcoidosis, there are significant interobserver differences that warrant more objective quantitative evaluation methods, which can be achieved by ML approaches [39].

It has been reported that deep CNN analysis can achieve superior diagnostic performance of sarcoidosis in comparison with the conventional quantitative analysis [40]. Moreover, it is known that myocardial scarring on CMR has a prognostic value in cardiac sarcoidosis patients [41]. A ML approach using regional CMR analysis predicted the combined endpoint of death, heart transplantation or arrhythmic events with reasonable accuracy in cardiac sarcoidosis patients [42].

### Ion channelopathies

Specific clinical and electrocardiographic markers have been associated with VT/VF occurrence in patients with Brugada syndrome. ML techniques can further improve the risk stratification performance of existing prediction models. Specifically, the combination of nonnegative matrix factorization and random forest models showed the best predictive performance compared with the random forest model alone and Cox regression models in this clinical setting [43]. Similarly, the random forest can better predict the occurrence of VT/VF post-diagnosis in congenital long QT syndrome in comparison with the conventional multivariate Cox regression model [44].

Furthermore, ML approaches can be used to explore the associations between genetic mutations and the occurrences of VAs triggered by ion channelopathies. As mutations of the SCN5A gene are known to be associated with Brugada syndrome and long QT syndrome, a study has applied ML methods to a list of missense SCN5A mutations and found mutations causing changes to the sodium current increase the risk of Vas [45]. However, the location, rather than the physicochemical properties of the mutation, is predictive, which highlights that functional studies remain important in this area of research [45]. As with Brugada and long QT syndromes, random forest analysis has been applied to identify important factors of ventricular arrhythmogenesis in catecholaminergic polymorphic VT [46].

### Drug-induced arrhythmias

Another interesting area pertinent to the implementation of ML techniques involves the prediction of drug-induced VAs. Specifically, using a support vector machine classifier, clustering by gene expression profile similarities showed that certain drugs prolong the QT interval in a limited number of patient groups [47]. As a result, ML methods may provide additional benefit in the current process of testing ion channel activities in the preclinical setting of cardiac safety assessment of drugs. Support vector machine has also been used for the prediction of Torsade des pointes of the different pharmacological agents [48]. It should be mentioned that ML methods have not only been implemented to identify a potential association between drugs and arrhythmic risk but also to identify moderators of the arrhythmic potential of specific medications [49]. Specifically, a study previously constructed a surrogate model for QT interval using multi-fidelity Gaussian regression and found that compounds blocking the rapid delayed rectifier potassium channels have the greatest QT-prolonging effect [50].

### Congenital structural heart disease

An integrated approach should be implemented in patients with complex congenital structural heart disease for the early prediction of adverse outcomes. For example, patients with Tetralogy of Fallot require risk stratification for the early identification of high-risk patients who require advanced healthcare management. ML techniques and specifically deep learning imaging analysis have been proposed to improve the risk stratification of Tetralogy of Fallot patients [51]. Using CMR data, a composite score of the enlarged right atrial area and depressed right ventricular longitudinal function identified a tetralogy of Fallot subgroup at increased risk of adverse outcome [51].

Furthermore, ML techniques have also studied in the prediction of postoperative arrhythmias following atrial septal defect closure. In this setting, a prediction model based on synthetic minority oversampling technique algorithm and the random forest was found to predict arrhythmias with excellent accuracy in a pediatric population [52]. This is further supported by Guo et al., which used a combination of ML techniques including support vector machine (SVM), random forest, naïve Bayes and adaptive boost to predict postoperative blood coagulation function for children with congenital heart disease [53]. The results offer promising evidence that ML models are more robust and accurate relative to traditional statistical methods.

### Arterial hypertension

Hypertension is a common condition that has been associated with adverse outcomes in the long-term setting. However, a prediction model is difficult to be constructed in young hypertensive patients mainly due to the lack of sufficient data in this population. Wu et al. used two ML methods, recursive feature elimination and extreme gradient boosting, to predict outcomes in young patients with hypertension [54]. The outcome was the composite of all-cause mortality, acute myocardial infarction, coronary artery revascularization, new-onset HF, new-onset atrial fibrillation/atrial flutter, sustained VT/VF, peripheral artery revascularization, new-onset stroke and end-stage renal disease [54]. While the proposed ML model was comparable with Cox regression for the measured outcome in the young patients with hypertension, it performed better than that of the recalibrated Framingham Risk Score model [54].

## Discussion

The role of ML algorithms for the risk stratification of VAs has been studied in different clinical settings. ML algorithms can provide an incremental value for the risk stratification of cardiomyopathies (HCM, ischemic and non-ischemic cardiomyopathy), cardiac sarcoidosis, channelopathies, congenital heart disease, arterial hypertension, as well as in predicting pharmacologically induced life-threatening arrhythmias.

The management and especially the prediction of life-threatening arrhythmias are paramount in clinical cardiology. A prediction model for VT one hour before its occurrence, using an artificial neural network, has been generated using 14 parameters obtained from heart rate variability and respiratory rate variability analysis [55]. ML techniques have been used to predict the occurrence of VAs using heartbeat interval time series. In this setting, the random forest model showed better performance using a length of heartbeat interval time series of 800 heartbeats, 108 s before the occurrence of arrhythmias [56]. These results can be implemented mainly in the prevention of cardiac arrest identifying high-risk patients prior to the occurrence of life-threatening VAs. Another study proposed a CNN algorithm to predict the onset of a VT using heart rate variability data [57]. The authors found that compared to other ML algorithms, the proposed one showed the highest prediction accuracy. Furthermore, ML algorithms have also been used for the prediction of VF. In this setting, QRS complex shape features were analyzed using artificial neural network classifiers [58]. This proposed model was found to achieve a better performance compared to the prediction accuracy using heart rate variability features [58].

Another area of ML implementation is in in-hospital monitoring. Timely and accurate discrimination of shockable versus non-shockable rhythms from external detectors and ICDs is of great clinical importance. Recently, the fixed frequency range empirical wavelet transform filter bank and deep CNN were used to analyze electrocardiographic signals [59]. The results showed excellent accuracy rates in classifying shockable versus non-shockable rhythms, VF versus non-VF and VT versus VF [59]. A deep learning architecture based on one-dimensional CNN layers and an LSTM network was found to be timely and accurate for the detection of VF in automated external defibrillators [60]. Furthermore, ML-based intensive care unit alarm systems have been found to achieve higher positive predictive values for the identification of asystole, extreme bradycardia, VT and VF compared to the bedside monitors used in the PhysioNet 2015 competition [9, 10, 61].

Except for life-threatening arrhythmias, ML algorithms have been used for the management of atrial fibrillation. Artificial intelligence-enabled electrocardiography was used to predict the incident atrial fibrillation [62]. In the same setting, ML algorithms have been implemented and outperformed conventional tools for the prediction of atrial fibrillation in critically ill patients who were hospitalized with sepsis [63]. In the field of invasive management of atrial fibrillation, ML-based classification of 12-lead ECG has been proposed as a useful tool for guiding atrial fibrillation ablation procedures and specifically in identifying patients suitable for pulmonary vein isolation alone vs. those needing additional ablation to pulmonary vein isolation [64]. Moreover, an ensemble classifier that used clinical and heart rate variability features were found to predict atrial fibrillation catheter ablation outcomes [65]. As a result, ML algorithms can have a role in the prevention and management of patients at risk or with documented atrial fibrillation, respectively.

However, ML algorithms present a series of limitations. Not only does ML require large sets of data during training, a considerable amount of time and resources is also necessary. Moreover, ML algorithms are susceptible to errors, such as mislabeled data, overfitting information and unavoidable bias [66]. Furthermore, ML techniques can only be trained to analyze a specific type of data; for example, although the random forest is highly effective in performing classification tasks, it is less effective when performing regression tasks as the algorithm cannot demonstrate precise, continuous nature predictions. Similarly, CNN is only effective in analyzing spatial patterns in images. Resultantly, different ML algorithms should be used for different purposes. Finally, ML algorithms shine a light on the debate regarding transparency, authority and other ethical ramifications. Therefore, an established framework that regulated the implementation of ML in clinical practice needs to be implemented [13].

## Conclusions

ML algorithms have been shown to ameliorate arrhythmic prediction performance in different clinical settings. However, it should be emphasized that prospective studies comparing ML algorithms to conventional risk models are needed while a framework is required prior to their implementation in clinical practice.

## Figures and Tables

**Table 1:** Summary of the baseline characteristics and the outcomes reported in ML studies provide data for risk stratification purposes

First author	Year of publication	Enrollment period	Number of patients	Age (years)	Males (%)	Type of clinical setting	Machine learning technique	Performance	Type of predicted arrhythmia	Features used for the prediction model
*Hypertrophic cardiomyopathy*										
Alis [24]	2020	2014–2019	64	48.13 ± 13.06	65.6	HCM	SVM, naïve Bayes, RF, k-NN	Accuracy SVM: 82.8% Naïve Bayes: 83.3% RF: 82.8% k-NN: 93.8%	Ventricular tachyarrhythmia	mean LGE%
Bhattacharya [19]	2019	Mean duration: 2.86 years	711	No VA: 54 ± 15 Yes VA: 49 ± 16	61.0	HCM	ensemble of logistic regression and native Bayes classifiers	Sens: 73%, spe: 76%, C-index = 0.83	Sustained VT, VF	22 features identified as predictive of VAr by the HCM-VAr-Risk Model
Lyon [25]	2018	N/A	123	47 ± 15	66.0	HCM	Density-based clustering algorithm	N/A	VA	Data from 12 lead ECG holter
Smole [20]	2021	N/A	2302	46 ± 19	62.9	HCM	RF, XGBoost, SVM, NN	Accuracy RF: 72% SVM (linear): 69% Bosted trees: 75% NN: 74% F1 score RF: 0.68 SVM (linear): 0.63 Boosted trees: 0.72 NN: 0,68	VT	demographic characteristics, genetic data, clinical investigations, medications and disease-related events
*Ischemic heart disease—heart failure—cardiomyopathies*										
Kotu [27]	2015	N/A	54	N/A	N/A	Post-myocardial infarction patients	K-NN, SVM, decision tree, RF	SVM classifier provided average accuracy of 92.6% and AUC of 0.921	VA	Quantitative discriminative features extracted from LGE-CMR image
Rogers [29]	2021	N/A	42	64.7 ± 13.0	97.6	Ischemic CMP	SVM, CNN	Accuracy of the SVM: 83.2% Accuracy of convolutional NN: 56.7%	VT/VF	Ventricular monophasic action potentials
Okada [28]	2021	2003–2015	122	60 ± 11	87.0	Ischemic CMP	SVM	Accuracy: 81% Correct classification: 86% NPP: 91%	VA	LGE-CMR data
Au-yeung [67]	2018	NA	788	60	77.3	HF	RF, SVM	Both RF and SVM methods achieve a mean AUC of 0.81 for 5-min prediction and mean AUC of 0.87–0.88 for 10-second prediction	Ventricular tachyarrhythmia	VA onset prediction with heart rate variability data from ICD
Meng [32]	2019	2017–2019	Retrospective: 500 Prospective: 1000	N/A	N/A	HFrEF	Information gain ranking, decision trees, logistic regression, SVM, RF, ANN	NA (study protocol)	VT/VF	Demographic, clinical, biological, electrophysiological, social and psychological variables
Wu [33]	2020	2003–2015	382	57 ± 13	72.0	HFrEF	RF	AUC: 0.88	VA	clinical heart failure course, baseline CMR imaging metrics, levels of the interleukin-6
Rocon [37]	2020	2011–2017	108	38.3 ± 15.5	48.1	Non-compaction CMP	SVD impute, Parameter Selection Algorithm, sequential forward selection, distance-weighted k-NN	Accuracy: 75.5% Sens: 77% Spe: 75%	Major adverse cardiovascular events	LVEF (by CMRI), RV end systolic volume (by CMRI), RV systolic dysfunction (by echo) and RV lower diameter (by CMRI)
*Implantable devices*										
Marzec [68]	2018	Minimum one year of observation	235	N/A	N/A	Patients with implantable electronic devices	Naïve, RF, decision tree analysis, k-NN, SVM	Accuracy Naïve: 74.5% RF: 76.6% Decision tree: 70.2% K-NN: 72.3% SVM: 74.5% F1 score Naïve: NA RF: 0.27 Decision tree: 0.13 K-NN: 0.00 SVM: NA	VT	Data about physical daily activities
Shakibfar [35]	2019	N/A	19,935	N/A	N/A	ICD patients	RF	Accuracy: 96% AUC: 0.80	VA (Electrical Storm)	ICD variables
*Channelopathies*										
Lee [43]	2021	1997–2017	516	50 ± 16	92.0	Brugada syndrome	Nonnegative matrix factorisation, RF	Random survival forest Precision: 83.4% Recall: 85.3% F1 score: 84.3% Nonnegative matrix factorization Precision: 87.1% Recall: 88.8% F1 score: 87.9%	VT/VF, Brugada syndrome	Clinical and electrocardiographic data
*Congenital heart disease*										
Dilller [51]	2020	10 year observation period	372	16.0	54.8	Tetralogy of Fallot	Deep learning algorithms	N/A	VA prognosis, cardiac death, cardiac arrest	Prediction of adverse outcome using CMR data
Sun [52]	2021	2009 – 2019	269	N/A	35.7	Following ASD closure in pediatric patients	RF, SVM, K-nearest neighbor, AdaBoost, decision tree	Accuracy k-NN: 78.9% Decision tree: 82.6% AdaBoost: 84.95% SVM: 89.3% RF: 94.7% AUC k-NN: 0.82 Decision tree: 0.80 AdaBoost: 0.75 SVM: 0.87 RF: 0.90	Postoperative arrhythmias	Demographic characteristics, cardiac imaging data and blood exams
*Different clinical settings*										
Sessa [49]	2020	January 2015–December 2016	18,018	74.8	39.9	Geriatric patients	Conditional inference tree	NA	VA	Specific medications
Taye [58]	2019	N/A	N/A	N/A	N/A	Public databases	ANN classifiers	Accuracy: 98.6% Sens: 98.4% Spe: 99%	Ventricular tachyarrhythmia	QRS complex shape
Okada [42]	2019	2000–2017	76	53 ± 10	59.0	Cardiac sarcoidosis	Supervised RF	Correct classification: 87% C-statistic: 0.91	VA, atrioventricular block	Regional scar burden
Taye [57]	2020	N/A	78	20.7–75.3	80.8	Spontaneous ventricular tachyar- rhythmia database	CNN, k-NN, ANN, SVM	Accuracy CNN: 84.6% ANN: 73.5% SVM: 67.9% k-NN: 65.9% AUC CNN: 0.78 ANN: 0.65 SVM: 0.63 k-NN: 0.62	Ventricular tachyarrhythmia	Heart rate variability signals
Wu [54]	2020	2012–2016	508	30.83 ± 6.17	75.0	Young hypertensive patients	Recursive feature elimination, extreme gradient boosting	C statistic: 0.757	Composite endpoint including sustained VT/VF	Demographics, medical history, vital signs, echocardiography, polysomnography, blood exams
Bergau [47]	2018	N/A	N/A	N/A	N/A	N/A	SVM	Sens: 85% Spe: 90%	Torsades de pointes	Gene expression differences
Chen [56]	2021	N/A	17	N/A	N/A	N/A	SVM, RF, XGboost	RF model with an average precision of 99.99% and recall of 88.98%	VA	Heartbeat interval time series
Yap [48]	2004	N/A	N/A	N/A	N/A	N/A	SVM, probabilistic NN, k-NN, decision tree	Accuracy SVM: 97.4% Probabilistic NN: 71.8% k-NN: 89.7% Decision tree: 38.5%	Torsade de pointes	Set of agents
Lee [55]	2016	2013–2015	N/A	N/A	N/A	N/A	ANN	Sens: 88.2% Spe: 82.4% PPV: 83.3% NPV: 87.5%	VT one hour prior to its occurrence	Heart rate variability and respiratory rate variability

HCM, hypertrophic cardiomyopathy; CMR, cardiac magnetic resonance; LGE, late gadolinium enhancement; VT, ventricular tachycardia; VA, ventricular arrhythmias; VF, ventricular fibrillation; SVM, support vector machine; SCD, sudden cardiac death; ANN, artificial neural network; HFrEF, heart failure with reduced ejection fraction; CMP, cardiomyopathy; ICD, implantable cardioverter-defibrillator; ASD, atrial septal defect; Sens, sensitivity; Spe, specificity; RF, random forest; CNN, convolutional neural network

## Data Availability

All data are included in the tables.

## Ready to submit your research? Choose BMC and benefit from:

- fast, convenient online submission
- thorough peer review by experienced researchers in your field
- rapid publication on acceptance
- support for research data, including large and complex data types
- gold Open Access which fosters wider collaboration and increased citations
- maximum visibility for your research: over 100M website views per year
